# Postpartum depression among women with pre-eclampsia and eclampsia in Tanzania; a call for integrative intervention

**DOI:** 10.1186/s12884-019-2395-3

**Published:** 2019-07-29

**Authors:** Barke Mbarak, Charles Kilewo, Saidi Kuganda, Bruno F. Sunguya

**Affiliations:** 1 Department of Obstetrics and Gynaecology, Muhimbili University of Health and Allied Sciences, 9 United Nations Road, 65001, Dar es Salaam, Tanzania; 2 Department of Psychiatry and Mental Health, Muhimbili University of Health and Allied Sciences, 9 United Nations Road, 65001, Dar es Salaam, Tanzania; 3 Department of Psychiatry and Mental Health, Muhimbili National Hospital, 65000, Dar es Salaam, Tanzania; 4 School of Public Health and Social Sciences, Muhimbili University of Health and Allied Sciences, 9 United Nations Road, 65015, Dar es Salaam, Tanzania

**Keywords:** Postpartum depression, Pre-eclampsia, Eclampsia

## Abstract

**Background:**

Postpartum depression (PPD) complicates maternal wellbeing, maternal-infant bonding, and cognitive function in children and woman’s relationship with her partner. Clinical observations suggest a higher risk of postpartum depression among those women with pre-eclampsia and eclampsia compared to the general population. However, the evidence is inconsistent and not from settings similar to Tanzanian. This study aimed to determine the magnitude and risk factors for PPD among women diagnosed with pre-eclampsia or eclampsia at Muhimbili National Hospital (MNH), Tanzania.

**Methods:**

This cross-sectional study was conducted among 390 women who had pre-eclampsia or eclampsia during pregnancy attending postnatal care clinic at MNH. PPD was assessed using Edinburg postnatal depression scoring scale (EPDS). Face to face interviews was conducted and data was analysed using descriptive and logistic regression analysis to address the two respective objectives.

**Results:**

PPD was prevalent among 20.5% of women who had pre-eclampsia or eclampsia but varied with severity. Factors associated with PPD included young age (AOR = 10.13 95% CI 1.99–52.02), being a single mother (AOR = 3.18 95% CI 1.02–9.95), having a lower level of education (AOR = 3.83 95% CI 1.45–10.16), having a perinatal death (AOR = 5.14 95% CI 2.53–10.45), lack of family support (AOR = 7.06 95% CI 1.25–39.90), and experience of stressful event during pregnancy (AOR = 15.14 95% CI 2.38–96.19).

**Conclusion:**

One in five women with pre-eclampsia or eclampsia had PPD and the magnitude increased with the severity of the disease condition. To address PPD, efforts should be done to screen and provide treatment to pregnant women presenting with pre-eclampsia or eclampsia, especially those with young age, low education level, single marital status, perinatal loss, lack of family support, and those reported to have a stressful event during pregnancy.

## Background

Postpartum depression (PPD) is one of the common complications of childbirth affecting approximately 10–15% of women globally [1]. Such a burden is high in low and middle income countries [2–5]. Tanzania is no exception. Although evidence is minimal, unpublished hospital based study in Tanzania reported the prevalence of PPD to be 16.2%.

Despite its devastating consequences to the woman, child, and family at large, PPD remains under-assessed and often misinterpreted [6]. If untreated, it can impair maternal functioning. It is also associated with poor nutrition and health of the infant; interference with breastfeeding, maternal-infant bonding, care of the infant and other children; and can affect the woman’s relationship with her partner [7, 8]. Moreover, evidence suggests an association between PPD with abnormal child development, cognitive impairment, and psychopathology [9–11].

The aetiology for postpartum depression has complex pathophysiology and is likely to result from the interplay of genetic, neuroendocrine and psychosocial factors [12]. Other factors that predispose to the development of postpartum depressive illness include a history of mood disorders, and a family history of psychiatric disorders, negative life events, poor marital relationships, lack of social support, drug abuse, maternal perfectionism and antenatal depression or anxiety [13, 14]. The risk for PPD has also been found to increase in women with pregnancy and delivery complications including pregnancy-related hypertension [15]. Pre-eclamptic women most often experience mood disorders which may have long term effects in comparison to women with uncomplicated pregnancy [16]. The evidence further suggests an increase in the risk of PPD with the severity of pregnancy induced hypertension [17, 18].

A woman with pre-eclampsia or eclampsia is more likely to be exposed to different maternal and neonatal complications which may affect her psychological wellbeing. Studies in developed countries have reported increased rates of PPD in women with a history of pre-eclampsia or eclampsia. Anecdotal and isolated clinical observations also suggest an increase in PPD among such high-risk patients in Tanzania. However, the evidence is not systematically collected to inform policies on such observation. This has led to missed opportunities in early detection and prevention of such devastating condition. This study, therefore, sought to examine the magnitude of PPD and associated risk factors following pre-eclampsia or eclampsia among women attending postnatal care clinic at MNH. This study addressed the two research questions. First, what is the prevalence of Postpartum Depression among women who had pre-eclampsia or eclampsia attending postnatal care clinic at MNH? Second, what are the associated risk factors of Postpartum Depression among women who had pre-eclampsia or eclampsia attending postnatal care clinic at MNH?

## Methodology

### Study design, site

This cross-sectional study was conducted in hospital settings of Muhimbili National Hospital (MNH) for six months from July 2016 to January 2017. MNH is the largest tertiary hospital in Tanzania, also serving as a referral hospital for high risk patients from other lower health facilities from Dar es Salaam and nearby regions. This is also designated as a training and teaching facility for Muhimbili University of Health and Allied Sciences (MUHAS). Care is provided by different cadres in the maternity unit under the supervision of specialist/consultant Obstetricians. MNH cares for on average about 1100 deliveries per month in a span of twelve months. Of these, 85 women are diagnosed with pre-eclampsia or eclampsia. Upon delivery, women diagnosed with pre-eclampsia or eclampsia are followed up in a postnatal clinic. This clinic serves an average of five women in a clinic day who had pre-eclampsia or eclampsia. There are four clinic days in a week, from Tuesdays to Fridays.

### Study population

We included all postpartum women who were diagnosed with pre-eclampsia or eclampsia during pregnancy; delivered and attended postnatal clinic (PNC) at MNH for follow up. The final diagnosis of patients with the hypertensive disorder in pregnancy was made at MNH by the specialists/consultants Obstetricians. Moreover, such diagnosis is usually written on the discharge summary note given to the patients when discharged home. In this study, the diagnosis of pre-eclampsia or eclampsia was obtained from the discharge notes mentioned above.

Data collection was set from two weeks post-delivery and span to six weeks. Enrolment was restricted to 4 weeks only because our postnatal clinic at MNH mostly attends women who are between two and six weeks post-delivery. After six weeks, most women are referred to other specialty clinics such as cardiac clinics and those with no complications are encouraged to attend lower level health facilities for care.

### Sample size and sampling

The sample size was calculated using the formula N = Z^2^P (1-P)/D^2^. Proportion (P) assumed to be 50% since there were no published studies in Tanzania or East Africa on PPD in women who had pre-eclampsia or eclampsia. With assumptions of a 95% confidence interval, 5% marginal error, the minimum sample size was estimated to be 384. However, we collected a sample of 390 mothers who fulfilled the selection criteria.

Convenience sampling technique was used to select participants. All women who had pre-eclampsia or eclampsia, attending PNC clinic at MNH were included until the required sample size was reached. Recruitment of participants was done at the clinic through discharge summary note that was given to them on discharge from the hospital, which had the diagnosis of the patient when she was admitted.

### Measurements

The outcome of interest was the Postpartum Depression measured using EPDS [19]. The 10-item EPDS explores events occurred in the immediate past 7 days. Each item has four choices scored from 0 to 3 from the first to last choice for item 1, 2 and 4. The scores are reversed from 3 to 0 for questions 3,5,6,7,8,9, and 10. The sum ranges from 0 to 30. Mothers who scored 13 and above are likely to be suffering from a depressive illness of varying severity [5, 19, 20]. Mothers with such scores were counselled and referred for further care.

Independent variables included psycho-socio, demographic, and obstetric characteristics. They included maternal age, marital status, level of education, occupation, family support, partner support, and quality of relationship with partner, financial status, history of mental illness, stressful events during pregnancy, parity, and gestation age at delivery, mode of delivery, birth weight of baby, neonatal admission and perinatal death [21–25]. Details on the obstetric information were obtained from both the discharge note and the mother.

Data on family support was obtained by asking the participant on how she perceived the assistance she received from family, including emotional, informational or material assistance. Data on the quality of the relationship with a partner was obtained using a question with three responses as very good, good or poor. Having a financial problem, this was defined as a situation where a woman could not afford spending money on necessities such as food or medication.

The stressful event was defined as any adverse emotional event that had occurred during pregnancy. Participants were asked if there was any stressful event such as a death in the family, divorce, accident, job loss, or any event that participant defined as stressful. Responses were categorized as yes or no.

The questionnaire used was translated before data collection, into Swahili with the help of a language expert, and then translated back to English to ensure consistency of the meaning.

Data were collected from participants with pre-eclampsia and eclampsia. Mild Pre-eclampsia was defined as new onset of hypertension (systolic blood pressure ≥ 140 mmHg or diastolic blood pressure ≥ 90 mmHg) and proteinuria (≥ 0.3 g. protein in 24 h urine specimen or ≥ 1+ using a urinary dipstick) after 20 weeks of gestation in a previously normotensive woman.

Severe Pre-eclampsia was characterized by systolic blood pressure ≥ 160 mmHg, diastolic blood pressure ≥ 110 mmHg at two occasions at least 4 h apart, with one or more complications such as thrombocytopenia, impaired liver function, renal insufficiency, pulmonary oedema, and new onset cerebral or visual disturbance. Eclampsia was defined as the presence of new-onset grandmal seizures in a woman with pre-eclampsia.

### Data collection

The finalized tool was pre-tested by the first author on a total of ten women. The Cronbach’s alpha for the EDPS was 0.89 showing adequate internal consistency. Only minor changes were done to improve the understanding of the other questionnaire items under the guidance of a psychiatrist, who is also a co-author.

A one-day training was conducted by the first author for the two research assistants who were also the nurse midwives working in maternity wards. They were trained on the purpose of the study, data collection techniques, and ethical issues pertinent to this study. Data were collected using face-to-face interviews after obtaining signed informed consent.

### Data analysis

All questionnaires were checked by the investigator for completion. Data were then entered, cleaned and analysed using descriptive and regression methods through Statistical Package for Social Sciences (SPSS) version 20.0. Four questionnaires were excluded due to missing data. Therefore, data of 386 participants were available for analysis. Results were expressed as frequencies and proportions for categorical variables and means and standard deviations for continuous variables. The prevalence of PPD was obtained as a percentage of women who scored ≥13 on EPDS. Chi-square test was used to find the differences in the proportion of PPD across all variables for socio-demographic, psychosocial and obstetric factors. Crude odds ratio (OR) with 95% confidence interval was calculated for all study variables. Multivariate logistic regression was then run to compute adjusted odds ratio (AOR) for variables with significant association to the dependent variable (*p* < 0.05) to determine the independent risk factors for PPD.

### Ethical considerations

The study was approved by the Research and Ethics Committee of the Muhimbili University of Health and Allied Sciences and permission to conduct the study was obtained from the executive director of MNH. Participation was voluntary and signed informed consent was obtained before data collection. Confidentiality was maintained throughout by ensuring that no names were used that can identify the participant. Participants that were found to be at risk of PPD and those who had suicidal thoughts were counselled and referred to a psychiatrist for further evaluation and treatment.

## Results

### The magnitude of postpartum depression

Seventy-nine out of 386 participants had an EPDS score of 13 and above. This category was considered as having PPD giving a prevalence of 20.5% (Table 1). The mean score on the EPDS was 7.82 ± 5.09, with scores ranging from 0 to 25. The magnitude of PPD was higher in eclampsia, and severe pre-eclampsia compared to mild pre-eclampsia (*p* < 0.001).

**Table 1:** Magnitude of PPD among participants (*N* = 386)

Diagnosis	Total N (%)	EPDS Score		*P* value
		< 13	≥13	
Mild pre-eclampsia	153 (100)	137 (89.5%)	16 (10.5%)	
Severe pre-eclampsia	170 (100)	127 (74.9%)	43 (25.3%)	
Eclampsia	63 (100)	43 (68.3%)	20 (31.7%)	< 0.001
Total	386 (100)	307 (79.5%)	79 (20.5%)	

### Characteristics of study participants

Majority of participants were between 20 and 34 years of age. Most of them were married or cohabiting, and about half of them were self-employed. Age, marital status and level of education showed significant association with PPD. Furthermore, all psychosocial variables showed significant association with PPD as well as some of the obstetric variables including parity and neonatal outcome (Table 2). None of the participants had pre-pregnancy history of depressive illness or any other psychological disorder.

**Table 2:** Characteristics of study participants (N = 386) and their association with postpartum depression following Pre-eclampsia or Eclampsia

Variable	Total N (%)	Postpartum Depression		*P* value
		Yes (%)	No (%)	
Age (years)				
(29.2 ± 6.28)^a^				
< 20	30 (7.8)	13 (43.3)	17 (56.6)	< 0.001*
20–34	269 (69.7)	58 (21.6)	211 (78.4)	
≥ 35	87 (22.5)	8 (9.2)	79 (90.8)	
Marital status				
Married/cohabiting	322 (83.4)	43 (13.4)	279 (86.6)	< 0.001*
Single/divorced/separated	64 (16.5)	36 (56.3)	28 (43.7)	
Level of education				
Lower level education	262 (67.9)	65 (24.8)	197 (75.2)	0.002*
Higher level education	124 (32.1)	14 (11.3)	110 (88.7)	
Occupation				
Housewife/unemployed	129 (33.4)	36 (27.9)	93 (72.1)	0.021*
Self employed	198 (51.3)	36 (18.2)	162 (81.1)	
Employed	59 (15.3)	7 (11.9)	52 (88.2)	
Parity				
Primipara	124 (32.1)	33 (26.6)	91 (73.4)	0.039*
Multipara	262 (67.9)	46 (17.6)	216 (82.4)	
GA at delivery				
Term	214 (55.4)	37 (17.3)	177 (82.7)	0.084
Preterm	172 (44.6)	42 (24.4)	130 (75.6)	
Mode of delivery				
Vaginal delivery	208 (53.9)	39 (18.8)	169 (81.2)	0.366
Caesarean section	178 (46.1)	40 (22.5)	138 (77.5)	
Neonatal outcome				
Livebirth	286 (74.1)	44 (15.4)	242 (84.6)	< 0.001*
Perinatal death	100 (25.9)	35 (35.0)	65 (65.0)	
Neonatal admission				
No admission	228 (59.0)	50 (21.9)	178 (78.1)	0.663
< 7 days	94 (24.4)	18 (19.1)	76 (80.9)	
≥ 7 days	64 (16.6)	11 (17.2)	53 (82.8)	
Birthweight (grams)				
< 2500	189 (49.0)	46 (24.3)	143 (75.7)	0.065
≥ 2500	197 (51.0)	33 (16.8)	164 (83.2)	
Family support				
Not satisfied	49 (12.7)	36 (73.5)	13 (26.5)	0.001*
Satisfied	337 (87.3)	43 (12.8)	294 (87.2)	
Partner support				
Not supportive	56 (14.5)	39 (69.6)	17 (30.4)	< 0.001*
Supportive	330 (85.5)	40 (12.1)	290 (94.5)	
Partner relationship				
Very Good	327 (84.7)	40 (12.2)	287 (87.8)	< 0.001*
Good	35 (9.1)	21 (60.0)	14 (40.0)	
Poor	24 (6.2)	18 (75.0)	6 (25.0)	
Financial problems				
Yes	61 (15.8)	39 (63.9)	22 (36.1)	< 0.001*
No	325 (84.2)	40 (12.3)	285 (87.7)	
Stressful event				
Yes	14 (3.6)	11 (78.6)	3 (21.0)^b^	< 0.001*
No	372 (96.4)	68 (18.3)	304 (81.7)	

Perinatal death – included women who had a stillborn baby or neonatal death

*GA* gestation age

*significant association (*p* < 0.05)

^a^mean age with standard deviation

^b^Fischer’s exact test used

### Factors associated with PPD

Table 3 shows the findings of logistic regression analyses of factors associated with PPD among women who had pre-eclampsia or eclampsia. Young, single women with lower education level and those who had perinatal death were more likely to have PPD. Moreover, those who lacked family support, and who reported having experienced stressful event during pregnancy had higher chances of having PPD as compared to those who had family support and did not experience any stressful event.

**Table 3:** Crude and adjusted odds ratio for the risk of PPD in women who had pre-eclampsia or eclampsia according to socio-demographic, obstetric and psychosocial variables (*N* = 79)

Variable	Depression N (%)	OR (95%CI)	Adjusted OR (95%CI)	*P* value
Age (years)				
< 20	13 (16.5)	7.55 (2.71–21.04)*	10.19 (1.99–52.02)	0.005
20–34	58 (73.4)	2.71 (1.24–5.94)*	5.7 (1.93–17.06)	0.002
≥ 35	8 (10.1)	1	1	
Marital status				
Married/cohabiting	43 (54.4)	1	1	
Single/divorced/separated	36 (45.6)	8.34 (4.63–15.04)*	3.18 (1.02–9.95)	0.047
Level of education				
Lower level education	65 (82.3)	2.59 (1.39–4.83)*	3.83 (1.45–10.19)	0.007
Higher level education	14 (17.7)	1	1	
Occupation				
Housewife/unemployed	36 (45.6)	2.88 (1.19–6.92)*	0.78 (0.23–2.67)	0.690
Self employed	36 (45.6)	1.65 (0.69–3.93)		
Employed	7 (8.9)	1		
Parity				
Primipara	33 (41.8)	1.7 (1.02–2.84)*	0.79 (0.33–1.90)	0.610
Multipara	46 (58.2)	1	1	
Neonatal outcome				
Livebirth	44 (55.7)	1	1	
Perinatal death	35 (44.3)	2.96 (1.76–4.99)*	5.14 (2.53–10.45)	< 0.001
Family support				
Not satisfied	36 (45.6)	18.9 (9.30–38.52)*	7.06 (1.25–39.99)	0.027
Satisfied	43 (54.4)	1	1	
Partner support				
Not supportive	39 (49.4)	16.6 (8.61–32.14)*	0.91 (0.05–16.29)	0.950
Supportive	40 (50.6)	1	1	
Partner relationship				
Very Good	40 (50.6)	1	1	
Good	21 (22.8)	10.8 (5.07–22.85)	0.71 (0.04–12.26)	0.810
Poor	18 (26.6)	21.5 (8.07–57.43)*	0.69 (0.52–9.26)	0.780
Financial problems				
Yes	39 (49.4)	12.6 (6.8–23.45)*	2.53 (0.79–8.03)	0.120
No	40 (50.6)	1	1	
Stressful event				
Yes	11 (13.9)	16.4 (4.45–60.34)*	15.14 (2.38–96.19)	0.004
No	68 (86.1)	1	1	

Perinatal death- included women who had a stillborn baby and neonatal death

*N* Number of participants, *OR* odds ratio, *CI* confidence interval

*significant association (*p* < 0.05)

## Discussion

The magnitude of PPD among women with pre-eclampsia and eclampsia was found to be 20.5% increasing with the severity of hypertension in pregnancy. The associated factors in our setting include age, marital status, level of education, stressful events during pregnancy, family and partner support.

The magnitude of PPD found in this study is slightly higher compared to the general population in the same region. This suggests an additional effect that may be attributed by psychopathological complications associated with pre-eclampsia and eclampsia. Studies conducted in different contexts reported an even higher magnitude of PPD [17, 18]. Although such a difference might be attributed to the contextual differences between Tanzania, Tehran, and the Netherlands, a different tool or cut-off point was also used. We used a more standardised questionnaire and recommended cut-off point for this condition [19].

Younger women diagnosed with pre-eclampsia or eclampsia were more likely to suffer from PPD compared to their older counterparts. Similar findings were also found in Canada, US [26–28]. However, the evidence is inconsistent on this regard in different settings such as Qatar, Brazil, and Cameroon [3, 21, 29]. Young maternal age has been associated with both increased risk of pre-eclampsia as well as PPD [30].

Single mothers were found to be at higher risk of getting PPD as compared to married mothers in our study. Single mothers may succumb to a number of psychosocial disadvantages. This may lead to a stressful situation, alter psychosocial stability, and therefore increased the risk of PPD. This finding is similar with evidence from Nigeria, Ghana, and India [31–33], but not to the body of evidence generated in US and Cameroon with regards to marital status [3, 27].

Similar to a number of studies [21, 28, 33, 34], we found a low level of education as a risk factor of PPD. Alternative or inconsistent findings were also reported elsewhere [3, 31, 35]. Low level of education is also associated with a number of social and economic disadvantages. Lack of awareness of some risky or danger signs including hypertension in pregnancy itself, self-care, among others may increase the risk of PPD. Moreover, the causal pathway for PPD is still unclear but the risk factors are known, and women with higher education level tend to be keen in following health advice given to keep themselves from possible risk factors.

Perinatal loss or any other loss for that matter is a risk for depression and other anxiety disorders [36]. In the context of hypertension in pregnancy, additional psychological trauma may further escalate the symptoms and lead to PPD. Our study found an association between such loss and PPD similar to studies in the Netherlands [18], Malaysia, and Bangladesh [37, 38]. Our study found associations between stressful events during pregnancy and PPD similar to other contexts [21, 39–41]. In the same pathway, even in the general population, having a stressful event is a trigger to depression. In addition to the trauma brought by pre-eclampsia or eclampsia and stress during pregnancy coupled with physiological and psychological changes, such risk factor pose a higher risk among the already vulnerable population.

Pregnancy and the postpartum period is an important period where the mother is generally provided with extra support by family members particularly, Tanzania is no exception. Lack of social support has been associated with increased risk of PPD in many studies conducted in different countries; Canada, Pakistan, China, Uganda [22–25, 41, 42]. The finding in our study is consistent with above studies where we found those women who were not satisfied with the family support they received, were at higher risk of PPD as compared to those who were satisfied with the family support they received during pregnancy and the postpartum period. These studies show that family support plays an important role in preventing PPD.

Evidence from this study should be interpreted carefully owing to the following three limitations. First, this was a cross-sectional study, therefore, we could not ascertain causality. However, our findings strongly correlate with those of other contexts. Second, Recall bias could also occur in this study, the disease state, pre-eclampsia or eclampsia, may add in the effect of recalling and therefore reducing confidence in answers to items asked. We tried to minimize this bias by providing quiet and ample time during the interview. Third, we conducted this study in a tertiary hospital and therefore, results may not be generalizable. Nevertheless, such results are consistent with other studies and may be useful in areas with similar context. Despite such limitations, this is the first study in our settings. So the results can be useful for planning and integrating PPD prevention and care package to women at risk.

## Conclusion

One in every five women with a history of pre-eclampsia or eclampsia had features suggestive of PPD in Dar es Salaam, Tanzania. The magnitude of PPD increases with the severity of the disease condition. Women who were young, single with a lower level of education or having perinatal death had a higher risk of postpartum depression. Addressing PPD in this and similar context, it is important to tailor interventions and target younger women, those with a lower level of education, having perinatal deaths or stressful events, as well as those with the challenging supporting system. Postpartum depression screening should be an integral part of postnatal care especially among women who had pre-eclampsia or eclampsia. Furthermore, Clinicians in maternal health should be aware of symptoms suggestive of PPD and be able to provide immediate care.

## Data Availability

The dataset used during and/or analyzed during the current study is not publicly available because it is still being used for other analyses in a research group. However, the dataset will be available from the corresponding author on reasonable request.
